# Multiple Amino Acid Supplementations to Low-Protein Diets: Effect on Performance, Carcass Yield, Meat Quality and Nitrogen Excretion of Finishing Broilers under Hot Climate Conditions

**DOI:** 10.3390/ani10060973

**Published:** 2020-06-03

**Authors:** Youssef A. Attia, Fulvia Bovera, Jinquan Wang, Mohammed A. Al-Harthi, Woo Kyun Kim

**Affiliations:** 1 Arid Land Agriculture Department, Faculty of Meteorology, Environment and Arid Land Agriculture, King Abdulaziz University, Jeddah 21589, Saudi Arabia; malharthi@kau.edu.sa; 2 Department of Veterinary Medicine and Animal Production, University of Napoli Federico II, via F. Delpino1, 80137 Napoli, Italy; 3 Department of Poultry science, University of Georgia, Athens, GA 30602, USA; jq.wang@uga.edu

**Keywords:** broilers, amino acid, low crude protein, carcass yield, nitrogen excretion, hot climate

## Abstract

**Simple Summary:**

Crude protein is an essential nutrient in poultry feed. Reducing the use of crude protein not only reduces the feed cost, but also minimizes pollution during poultry production. Thus, finding the minimum protein requirement in broiler diet without compromising broiler growth is the objective in the present study. Supplementing essential amino acids including methionine and lysine to the low-protein diet showed comparable growth performance and carcass yield to the regular protein diet. Thus, reducing the crude protein level is possible if the essential amino acid balance is adequate for broiler growth.

**Abstract:**

The objective of this study was to evaluate the effect of low-protein diets with amino acid supplementation on growth performance, carcass yield, meat quality and nitrogen excretion of broilers raised under hot climate conditions during the finisher period. In trial 1, broilers from 28 to 49 days of age were fed 18% crude protein (CP) as a positive control or 15% CP supplemented with (1) DL-methionine (Met) + L-lysine (Lys), (2) Met + Lys + L-Arginine (Arg), or (3) Met + Lys + L-Valine (Val). In trial 2, broilers from 30 to 45 days of age, were fed an 18% CP diet as a positive control or 15% CP supplemented with Met, Lys, Arg, Val, L-Isoleucine (Ile) or combination with glycine (Gly) and/or urea as nitrogen sources: (1) Met + Lys, (2) Met + Lys + Arg, (3) Met + Lys + Val, (4) Met + Lys + Ile, (5) Met + Lys + Arg +Val + Ile + Gly, and (6) Met+ Lys + Arg + Val + Ile + Gly + urea. Protein use was improved by feeding low-protein amino acid-supplemented diets as compared to the high-protein diet. Feeding 15% crude protein diet supplemented with only methionine and lysine had no negative effects on carcass yield, CP, total lipids and moisture% of breast meat while decreasing nitrogen excretion by 21%.

## 1. Introduction

In hot climates, the growth of broilers is usually slow, as high temperature adversely affects feed intake, and high-protein diets, used to sustain animal growth, may impair broiler tolerance to heat stress due to the high heat increment induced by protein metabolism [1,2]. Decreasing dietary protein levels of broiler diets could be a good strategy, but the amino acid requirements for broiler chicks must be satisfied by supplementing synthetic amino acids to the basal diets. In this way it is possible to achieve several goals: (1) minimize the excess of amino acid input and thus nitrogen pollution; (2) improve the bird tolerance to hot climate; (3) obtain satisfactory growth performance; all this can increase the profit for the poultry industry [3,4,5].

Environmental pollution due to nitrogen excretion is an important issue in poultry houses and results in negative impacts on the health of workers and birds, soil, and ground water [4,5]. Thus, reducing dietary protein levels with proper amino acid supplementation has been of great interest [2,4,6,7,8,9,10,11,12,13,14]. It is well recognized that methionine (Met) is typically the first limiting amino acid in practical broiler diets, whereas lysine (Lys) is the second in broiler diets. However, this depends on the ingredient composition of the diets [4,15,16]. There are several commercially available synthetic amino acids, such as methionine, lysine, threonine (Thr), tryptophan (Trp), and arginine (Arg) [4,12,17] that can be used to fulfill amino acid requirements in low-protein diets. 

Fancher and Jensen [6], feeding female broilers on low-protein, corn/soybean meal diets supplemented with synthetic amino acids from 3 to 6 weeks of age, found worse performance in comparison to a higher-protein finisher diet. Liu et al. [18] reported that a decrease in protein level from 22.5% to 20% impaired body weight gain from 1 to 21 days of age. Along the same line, Holsheimer and Janssen [19] reported that Thr, Trp and Arg were limiting in diets containing 17% CP supplemented with Met and Lys when compared to 20% CP diet during 3–7 weeks of age. They also indicated that 0.77% Thr and 0.22% Trp were enough in finisher broiler diets during 3–7 weeks of age. Han et al. [7] observed that broiler chicks fed low-protein diets (16% CP) supplemented with Met, Lys, Arg, valine (Val), and glutamic acid during 3-6 weeks of age had high body weight and better feed conversion ratio (FCR) than those fed a 20% CP diet while maintaining similar body fat content. Moreover, Laudadio et al. [8] indicated that female broiler chicks fed 17% CP diet supplemented with Met plus cysteine (Cys) showed higher body weight than chickens fed 20% CP during 3–7 weeks of age. Lipstein et al. [20] and Lecercq et al. [21] reported that low-protein diets supplemented with Met and Lys prevented increased carcass fat deposition during the finisher period. However, severely low crude protein diets may increase abdominal fat deposition in carcass [22,23]. A 3% reduction in CP compromised body weight and feed conversion during 21–42 day of age but the supplementation of Val+Ile (isoleucine)+Arg+Gly was able to restore both [24]. 

Broiler chicks consume the highest proportion (~60%) of their feed during the finisher period, when they are more susceptible to heat stress because of their bigger body size. However, few studies have been performed to evaluate the effects of low-protein diets supplemented with multiple key synthetic amino acids on growth performance and carcass yield, under hot climate during finisher period. Thus, the objective of this study, organized in two different trials (Trial 1 and 2) was to evaluate the effect of low-protein diets supplemented with different levels of various essential amino acids on body weight gain, feed conversion ratio, nitrogen excretion, carcass yield, and inner organ weight of broilers during the finisher period and farmed under hot climate conditions.

## 2. Materials and Methods 

### 2.1. Animal Husbandry:

Broiler chicks were reared in battery-brooders (35 cm length × 25 cm width × 30 cm height) with 23:1 light-dark cycle at day 1, gradually reduced to 18:6 light-dark cycle. Birds were fed from tube feeders and drank from automatic nipple drinkers. Mash diets and water were offered ad libitum throughout the trials. Birds were vaccinated against Newcastle disease virus with Hatchner (B1) at 7 days old and Lasota at 20, 30 and 45 days of age. The average indoor ambient temperature and relative humidity were (mean ± standard deviation): 34 ± 6 °C and 54 ± 9% in Trial 1 and 35 ± 5 °C and 57 ± 11% in Trial 2, respectively. The experimental protocol was approved by the Animal and Poultry Production Scientific and Ethics Committee of Damanhour University. The care and handling of the animals were performed so as to maintain their rights, ensure their welfare, and cause minimal stress, according to International Guidelines for research involving animals (Directive2010/63/EU).

### 2.2. Experimental Design and Diets

Trial 1 comprised 7 groups (each with 25 Hubbard male broilers, 5 replicates of 5 birds) which had the same feed ingredients and only differed in the protein source and the type of amino acid included. Two different CP diets were formulated based on the protein source: vegetable diets with corn soybean meal (groups 2, 3 and 4) or animal protein diets with fish and meat meals (groups 5, 6 and 7). From 28 to 49 days of age, the groups were submitted to the following dietary treatment: 18% CP (group 1); 15% CP supplemented with Met and Lys (group 2 and group 5); 15% CP supplemented with Met, Lys and Arg (group 3 and group 6); 15% CP supplemented with Met, Lys, Arg and Val (group 4 and group 7). In trial 1, the amounts of Met and Lys supplemented were 0.20% and 0.28% for vegetable diets (group 2, 3 and 4), and 0.19% and 0.26% for animal diets (groups 5, 6 and 7), respectively. The supplemented amounts of Arg, Val and Ile were 0.17%, 0.15% and 0.0% for the vegetable protein diet (group 2, 3 and 4), and 0.22%, 0.15% and 0.05% for the animal protein diet (groups 5, 6 and 7), respectively.

Trial 2 comprised 7 groups (25 male Hubbard broilers, 5 replicates of 5 birds per group) which had the same feed ingredients and only differed in the type of amino acid included. From 30 to 45 days of age, the groups were submitted to the following dietary treatments: group 1 as positive control with 18% CP; 15% CP supplemented with Met at 0.19%, Lys at 0.25% (group 2); 15% CP supplemented with Met at 0.19%, Lys at 0.25% and Arg 0.21% (group 3); 15% CP supplemented with Met at 0.19%, Lys at 0.25% and Val at 0.10% (group 4); 15% CP supplemented with Met at 0.19%, Lys at 0.25% and Ile (group 5); 15% CP supplemented with Met at 0.19%, Lys at 0.25%, Arg at 0.21% Val at 0.10%, Ile and Gly as an amino nitrogen source (group 6); and 15% CP supplemented with Met at 0.19%, Lys at 0.25%, Arg at 0.21% Val at 0.10%, and Ile (as a nitrogen source to equalize nitrogen content between the positive control diet and the 15% CP diet; group 7). 

The diets (Trial 1 and 2) were formulated based on Nutrient Requirements of Poultry from National Research Council (NRC) [25] tabulated values for feedstuffs (Table 1). Met and Lys levels of the low protein 15% CP diets were equal to those of the positive control diet. Amino acids were supplemented and analyzed by Evonik (Degussa-Hüls AG, Frankfurt am Main, Germany). Amino acids were supplemented to meet NRC amino acid requirements [25] for broiler chicks.

### 2.3. Growth Performance and Carcass Yield

Chicks were weighed at the beginning, at 28 days of age in Trial 1 and at 30 days in Trial 2, and at the end at 49 days of age for Trial 1 and 45 days of age for Trial 2. Average initial body weights in Trial 1 and 2 were (mean ± standard deviation) 1,008 ± 10 g and 1,164 ± 12 g, respectively. In both trials there were no differences among groups for initial body weight and the Body Weight Gain was calculated as the difference between final and initial body weight. Additionally, for both trials, the amounts of administered and residual feed were weighed on the same day as the weighing of the birds, and total feed and protein intakes were calculated as the difference between administered and residual feed. Feed and protein conversion ratios were calculated by dividing feed and protein intake by body weight gain. In Trial 2, at 40 days of age, represented samples (500 g) of excreta (n = 5 per treatment) as one sample per replicate were collected and cleaned from feathers, feeds and scabies, and then moisture and nitrogen contents were determined according to the Association of Official Agricultural Chemists (AOAC) [26]. A total of 35 broilers at the end of Trials 1 and 2 were slaughtered for carcass evaluation. In Trial 1, moisture, crude protein, ether extract, and ash of the skinless boneless breast meat (pectorals major) from the slaughtered birds were further determined in pooled triplicate samples according to AOAC [26]. The crude protein, ether extract, and ash content were divided by dry weight for breast meat chemical characteristics. 

### 2.4. Statistical Analysis:

Data from each trial were analyzed using the general linear model procedure of Statistical Analysis Software [27] using a one-way analysis of variance. The experimental unit was the replicate. Mean differences were tested using student Newman Keuls Test [27] to evaluate the differences among means at *P* ≤ 0.05.

## 3. Results

In general, broilers in both trials showed signs of heat stress, including panting, lying down, and flapping their wings to dissipate heat, but differences in behavior responses were not quantitative among different treatments (data not shown).

In Trials 1 and 2, during the whole finishing period, there were no significant differences among groups for body weight, weight gain, feed intake and feed conversion ratio (Table 2 and Table 3).

However, in both trials the 15% CP diet groups with amino acid supplementation showed lower (*P* < 0.05) protein intake and protein conversion ratio (PCR) compared to group 1 (Table 2 and Table 3).

In Trial 1, there were no differences among groups for carcass yield, body organs and breast meat chemical compositions (Table 4). In Trial 2 (Table 5), the supplemented groups 2, 3 and 4 showed a significantly higher (*P* < 0.05) gizzard percentage compared to groups 1 and 7. Groups 2, 3 and 7 showed a lower (*P* < 0.05) spleen percentage compared to group 1 (Table 5). However, there were no differences among the other carcass traits.

## 4. Discussion

### 4.1. Growth Performance, Feed/Protein Conversion Ratio and Nitrogen Excretion of Broiler Chicks

Our previous studies showed that broiler growth was low at high temperature (34 ± 6 – 35 ± 5 °C and 54 ± 9 and 57 ± 11% Relative Humidity) [1,2]. In the present study, the results from Trials 1 and 2 indicated that reducing the CP level from 18% to 15% with the supplementation of amino acids under hot climates and during the finisher period reduced the overall protein intake of broilers without detriment to growth performance. In both trials, the low final body weight compared to Attia et al. [28] could be attributed to the high ambient temperature and humidity (34 and 35 °C and 54% and 57%, respectively). Heat stress in tropical and subtropical regions not only causes economic effects in the form of retarded growth performance, but also induces animal health and welfare concerns [29]. The hot temperatures during the daytime retarded the growth of 7-week-old broilers under 2 kg in both trials. Extreme environmental conditions should be avoided in general through ventilation or reducing stock density; however, hot climate conditions are unavoidable in tropical areas. The results in the current study indicate that satisfactory broiler growth and feed conversion could be achieved with a reduction by 16.6% (from 18% to 15%) of the crude protein content of the diet in finishing broilers raised under hot climate conditions, but the low-protein diet must be supplemented with adequate amounts of Met and Lys, and the same results were also obtained with only vegetable or partially animal protein sources in the diet. 

Several studies have suggested that reducing protein levels in diets with adequate supplementation of indispensable amino acids will not compromise broiler growth performance during the finisher period [10,11,12,13,14]. Recent research indicates that in broilers from 1 to 35 days of age, the CP of the diets can be reduced from 210 to 180 g/kg without detriment to broiler performance, but further reduction from 180 to 165 g/kg of diet CP worsened FCR, although the level of essential amino acids was maintained [30]. Additionally, van Harn et al. [31] observed that, in broilers from 1 to 35 days of age, during the finisher period, the crude protein of the diets could be reduced by 2.2–2.3 percentage points (from 19.8%) without negative effects on bird performance if the essential amino acids were correctly balanced, but further decrease could penalize some carcass traits. Chrystal et al. [30] attributed these results to a possible deficiency of non-essential amino acids such as glycine and serine at the lowest level of diet CP. As animal age increases in the present trial (28–49 and 30–45 day of age for Trials 1 and 2, respectively), the requirements of birds for non-essential amino acids could be satisfied even while reducing the protein content of the diet to 15%. The retarded growth performance under hot climate also contributed to the change in requirements. However, the current results could not distinguish the confounding effects of heat stress and reduction of protein. It is interesting to observe that, under hot climate conditions, reducing the CP level to 15% with key amino acid supplementation can maintain the same growth performance of finishing broilers as 18% CP.

Arginine or Valine supplementation in 15% CP animal/vegetable diets over Met and Lys did not show any additive impact in growth performance. In the literature, the availabilities of Val and Arg in fish meal were low (67.3% and 62.6%, respectively); thus, supplementation of Val and Arg was necessary to maintain optimum growth performance in the fish meal-containing diet [32,33]. Wide variations in amino acid digestibility have been reported in animal source proteins, particularly in terms of batch-to-batch differences [34]. The differences in responses to Arg or Val addition may be due to the difference in dietary protein sources, inclusion levels and amino acid requirements for broiler males and females [35,36,37,38]. It has been suggested that the protein and/or amino acid levels of the diets, strain, and the age of the birds may affect the response to the level of amino acid addition [7,39]. The supplementation of Met, Lys, Thr, Arg and Val was found to improve the growth of broilers [39], and the supplementation of Met, Lys, Arg, Val and Thr plus glutamic acid to 16% CP diet during 3–6 weeks of age also increased growth performance [36].

In the 14% CP soybean meal diet, the limiting amino acids were Met, Lys, Thr and Val [40], whereas Met, Lys, Thr, Arg, Val and Ile were limited in the 12% CP diet [41]. It has also been reported a low-protein diet (17%) with balanced Lys, Met, Thr and Trp levels had the same performance as a 19% CP diet during 22 to 56 days of age [42,43]. However, the present research indicates that 15% CP diet supplemented with Met + Lys showed no significant differences in growth and FCR compared to the positive control diet. Therefore, our trial suggests that limiting amino acids are related to CP percentage, environmental temperature and ingredient composition (availability of amino acids) of the experimental diets. 

In the present study, soybean meal, a high-quality protein ingredient [34], has been used as primary protein source. The lack of response to Ile in Trial 2 indicated that this amino acid is not limiting in 15% CP diet under heat stress and/or there might be the antagonism between Ile and leucine, because both are branched-chain amino acids [17,44,45]. This inconsistency among many studies may be attributed to diet composition, amino acid content and availability in ingredients, age of birds, and environment conditions (temperature and humidity). 

Glycine as a source of amino nitrogen or urea, as a nitrogenous source had no additive influence on the growth of broilers in Trial 2, indicating that the 15% CP diet supplemented with Met and Lys furnishes adequate amounts of dispensable amino acids for broiler growth during 30–45 days of age under a hot climate. Awad et al. [46] reported that the addition of glycine did not compensate the depressed growth performance in broilers under hot climate. The lack of responses to glycine as a nonessential amino acid on improving broiler performance is in contrast to the finding of Dean et al. [47]. Supplementation of glycine enhanced growth performance of broilers fed a low protein amino acid-supplemented diet because low-protein diets are low in nitrogen sources for making non-essential amino acids and other essential nitrogen compounds in the body [47]. Urea in non-ruminant animals had no nutrient values [48,49]. Thus, further studies are needed to evaluate the amino acid requirements under different conditions involving ingredients and rearing conditions.

The present results indicated that the diet containing 15% of CP supplemented with Met and Lys may satisfy all amino acid requirements for optimal broiler growth under hot climate. Some studies have shown that broilers eat more feed to satisfy their protein or amino acid needs under hot climate [9,50,51]. The feed conversion ratios in this study are similar to other studies showing that there were no significant differences in feed intake and efficiency of feed use among birds fed a low protein and a high-protein diets [8,51,52,53].

The present results indicated that protein intake was high, and protein/gain ratio was poorer in the 18% CP group than the groups fed low-protein amino acid-supplemented diets. Aletor et al. [50] indicated that a lower dietary protein level improves the protein use due to the lower protein turnover rate. There was a numerical 21.1% reduction in nitrogen excreted when 15% CP supplemented with Met and Lys in the present study. These results are in agreement with several studies reporting that low-protein amino acid-supplemented diets had no negative effect on broiler performance, while reducing nitrogen excretion by 33.6% [24,43,52,54,55]. The reduction of nitrogen excretion also influences ammonia volatilization in poultry houses: supplementing amino acids in low-protein diets reduced ammonia volatilization by 45% in poultry [4,5,56].

In general, due to the limits of the experimental conditions, it is not clear that the amino acid requirement change was triggered by heat stress or a decrease on performance or both; thus, further research is needed. 

### 4.2. Carcass Yield, Body Organs and Chemical Composition of Breast Meat

Carcass yield results in the current study indicate that Met and Lys supplementation in 15% CP diet provides sufficient amount of amino acids for maintaining optimum growth and development of body organs compared to 18% or 18.5% CP diets under hot climate. Alleman and Leclercq [51] reported that reducing CP content from 20% to 16% during 23–44 days of age had no negative effect on breast muscle percentage. However, several researchers have concluded that lowering the protein level in diet, maintaining appropriate levels of essential amino acids (Arg, Ile, Val and Trp) allowed normal protein gain, but increased fat deposition in both fat and lean lines of broilers, revealing that protein or lipid deposition was potentially controlled independently [8,57,58].

In agreement with the present results, Aletor et al. [50] found that decreasing dietary CP percentage while satisfying the amino acid requirements of broilers had no detrimental effect on relative weights of breast or thigh, which is similar to the present findings. However, in contrast to the present results, there were significant increases in the relative weights of liver and abdominal fat which may indicate higher lipogenetic activity in the body. Other studies have shown a significant increase in abdominal fat deposition when broilers or ducks were fed low-CP diets [22,23,59]. However, according to the current study, under hot climates, broilers may use amino acid and protein from low-CP diets better than those under the normal temperature conditions.

The gizzard and spleen percentage differences were unexpected in the current study. Further studies are necessary to explain this change. The protein level in finisher diets for broiler chickens could be reduced from 18% to 15% without adverse effect on broiler growth performance during 28–49 days of age under hot climates if Met and Lys are supplemented to meet requirements while decreasing nitrogen excretion by 21%. Therefore, protein use and economic efficiency in birds fed 15% CP diet supplemented with Met and Lys were improved because dietary protein input was lower compared to the high-protein control diet. Furthermore, the non-significant influences on carcass yield, abdominal fats, and total lipids of meat indicated that these traits are not compromised feeding the low-protein diet supplemented with Met and Lys.

## 5. Conclusions

Under hot climate conditions and during the finisher period, feeding a low-protein diet fortified with essential amino acids improved protein use and deceased nitrogen excretion without negative effect on performance and carcass yield of broiler. Thus, in the above-mentioned environmental conditions, the protein level of the finishing broiler diets can be reduced from 18% to 15% with an adequate supplementation of only methionine and lysine.

## Figures and Tables

**Table 1:** Composition, and calculated and analyzed nutrients of diets in trial 1 and 2.

Ingredients, %	Trial 1			Trial 2	
	18%	15% Animal	15% Plant	18%	15%
Yellow corn	71.63	73.00	73.00	66.86	72.40
Soybean meal	21.00	15.00	20.00	24.71	16.90
Fish meal (72% CP herring)	2.00	1.73	0.00	2.00	2.00
Meat meal	2.00	1.73	0.00	0.00	0.00
Soybean oil	0.50	1.45	1.70	1.65	1.56
Bone meal	1.40	1.68	2.38	1.70	1.88
Lime stone	0.82	0.72	0.63	0.75	0.72
Vit + Min premix ^1^	0.25	0.25	0.25	0.25	0.25
NaCl	0.30	0.30	0.30	0.30	0.30
DL-methionine	0.10	0.19	0.20	0.11	0.19
L-lysine	0.00	0.26	0.28	0.06	0.25
Sand	0.00	3.69	1.26	1.61	3.55
Total	100.0	100.0	100.0	100.0	100.0
Calculated values, %					
ME kcal/ kg	3008	3000	3041	3000	3004
Tryptophan	0.23	0.18	0.19	0.24	0.19
Ca	1.01	1.01	1.01	0.91	0.91
Available P	0.41	0.41	0.41	0.37	0.37
Determined values ^2^, %					
Crude protein	19.04	15.54	15.18	18.63	15.23
Methionine	0.30	0.25	0.24	0.31	0.26
TSAA	0.92	0.70	0.68	0.59	0.51
Lysine	0.92	0.70	0.72	0.96	0.74
Arg	1.02	0.84	0.81	1.03	0.86
Val	0.89	0.69	0.71	0.82	0.71
Ile	0.71	0.62	0.60	0.73	0.59

^1^ Premix provides per kg of diet: vitamin A, 8000 international units (IU); vitamin E, 9 mg; menadione (as menadione sodium bisulfite), 150 IU; Vit. D3, 1,000 ICU; riboflavin, 4.0 mg; Ca pantothenate, 10 mg; nicotinic acid, 12 mg; choline chloride, 300 mg; vitamin B12,2 mg; vitamin B6, 1.2 mg; thiamine (as thiamine mononitrate), 2.0 mg; folic acid, 40 mg; d-biotin, 0.05 mg. Trace minerals (mg per kg of diet): Mn, 75; Zn, 40; Fe, 40; Cu, 3; Se, 0.15; iodine, 0.8 and 500 mg an antioxidant. ^2^ Calculated based on the analyzed amino acids of feed ingredients except for tryptophan, which was not determined.

**Table 2:** Growth performance from 28 to 49 day of age (Trial 1).

Item	Groups							SEM ^1^	*P* Value
	1	2	3	4	5	6	7		
Body weight at 28 d, g	1009	1006	1011	1005	1002	1018	1006	15.3	0.99
Body weight at 49 d, g	1964	1940	1970	1938	1947	1970	1853	28.7	0.88
Body weight gain, g	955	934	959	933	945	952	947	21.1	0.78
Feed intake, g	2212	2223	2257	2263	2223	2226	2226	12.7	0.15
Protein intake, g	409.2 ^a^	333.4 ^b^	338.5 ^b^	339.4 ^b^	333.5 ^b^	333.9 ^b^	333.9 ^b^	1.96	0.01
Feed conversion ratio, g/g	2.32	2.38	2.35	2.43	2.35	2.34	2.35	0.11	0.74
Protein Conversion Ratio, g/g	0.428 ^a^	0.357 ^b^	0.353 ^b^	0.364 ^b^	0.353 ^b^	0.351 ^b^	0.353 ^b^	0.009	0.01

^a,b^: Means within a row not sharing a common a superscript differ significantly *P* ≤ 0.05, based on Duncan’s test; ^1^ SEM, standard error of the mean.

**Table 3:** Growth performance from 30 to 45 day of age (Trial 2).

Item	Groups							SEM	*P* Value
	1	2	3	4	5	6	7		
Body weight 30 d, g	1155	1176	1167	1153	1171	1160	1166	25.1	0.99
Body weight 45 d, g	1959	1977	1992	1988	1981	1993	1971	37.2	0.99
Body weight gain,	804	801	824	835	810	834	806	25.8	0.95
Feed intake, g	1717	1756	1763	1782	1796	1763	1753	23.4	0.38
Protein intake, g	309 ^a^	263 ^b^	264 ^c^	267 ^b^	269 ^b^	265 ^b^	263 ^b^	3.05	0.01
Feed conversion ratio, g/g	2.14	2.19	2.14	2.13	2.22	2.11	2.17	0085	0.98
Protein conversion ratio, g/g	0.384 ^a^	0.329 ^b^	0.321 ^b^	0.320 ^b^	0.333 ^b^	0.317 ^b^	0.326 ^b^	0.014	0.01
Excreta nitrogen, %	4.03	3.18	3.25	3.47	3.79	3.71	3.90	0.54	0.17
Excreta dry matter, %	21.0	20.9	21.8	21.1	21.6	22.2	22.7	1.6	0.46

^a–c^ Means within a row not sharing a common a superscript differ significantly *P* ≤ 0.05, based on Duncan’s test.

**Table 4:** Carcass yield, body organs percentage and breast meat chemical composition (Trial 1).

Item	Groups							SEM	*P* Value
	1	2	3	4	5	6	7		
Carcass yield									
Dressing ^1^, %	62.2	61.9	62.2	62.0	63.2	63.4	62.5	0.97	0.17
Breast + wings, %	23.4	23.3	22.5	24.4	23.6	22.4	22.1	0.77	0.40
Thigh + legs, %	21.7	22.3	22.0	21.6	21.6	23.6	22.6	0.67	0.40
Abdominal fat, %	1.71	1.82	1.83	1.81	1.80	2.19	1.93	0.24	0.36
Liver, %	2.42	2.67	2.24	2.18	2.14	2.31	2.29	0.44	0.10
Heart, %	0.64	0.65	0.64	0.69	0.62	0.65	0.65	0.041	0.93
Pancreas, %	0.23	0.25	0.22	0.21	0.24	0.24	0.23	0.012	0.43
Spleen, %	0.131	0.125	0.108	0.161	0.125	0.134	0.149	0.013	0.14
Chemical characteristics of breast meat									
Moisture, %	74.2	74.4	73.8	75.1	74.9	74.6	74.9	2.42	0.58
Crude protein, %	82.7	81.4	81.4	82.0	81.9	82.0	82.0	1.73	0.74
Ether extract, %	13.1	13.4	13.8	13.0	13.2	13.6	13.5	0.74	0.68
Ash, %	4.2	5.2	4.8	5.0	4.9	4.4	4.5	0.12	0.39

^1^ Head, giblets, feet and eviscerate were not included.

**Table 5:** Carcass yield of broiler chickens (Trial 2).

Item	Groups							SEM	*P* Value
	1	2	3	4	5	6	7		
Dressing ^1^, %	62.4	63.7	61.8	60.8	61.2	63.2	60.0	0.96	0.11
Breast + wings, %	30.5	31.1	30.2	29.2	30.4	32.4	29.6	0.71	0.07
Thigh + legs, %	32.9	34.8	32.1	32.1	31.7	31.8	31.4	0.89	0.16
Abdominal fat, %	1.71	1.80	2.10	1.84	1.45	1.33	1.62	0.25	0.39
Liver, %	1.84	1.95	2.22	2.09	2.02	1.93	1.99	0.13	0.49
Heart, %	0.44	0.46	0.51	0.47	0.42	0.44	0.45	0..03	0.43
Gizzard, %	1.54 ^b^	2.00 ^a^	1.90 ^a^	1.88 ^a^	1.83 ^a,b^	1.76 ^a,b^	1.53 ^b^	0.10	<0.01
Pancreas, %	0.21	0.18	0.20	0.19	0.20	0.18	0.19	0.015	0.69
Spleen, %	0.129 ^a^	0.083 ^c^	0.087 ^b,c^	0.124 ^a,b^	0.115 ^a–c^	0.121 ^a,b^	0.088 ^b,c^	0.012	0.03

^a–c^ Means within a row not sharing a common a superscript differ significantly *P* ≤ 0.05, based on Duncan’s test; ^1^ Head, giblets, feet and eviscerate were not included.
